# *Cordyceps militaris* (L.) Link Fruiting Body Reduces the Growth of a Non-Small Cell Lung Cancer Cell Line by Increasing Cellular Levels of p53 and p21

**DOI:** 10.3390/molecules200813927

**Published:** 2015-07-31

**Authors:** Ana Bizarro, Isabel C. F. R. Ferreira, Marina Soković, Leo J. L. D. van Griensven, Diana Sousa, M. Helena Vasconcelos, Raquel T. Lima

**Affiliations:** 1 Laboratory of Microbiology, Department of Biological Sciences, Faculty of Pharmacy, University of Porto, Rua Jorge Viterbo Ferreira 228, Porto 4050-313, Portugal; E-Mails: sofiabizarro@gmail.com (A.B.); dsousa@ipatimup.pt (D.S.); hvasconcelos@ipatimup.pt (M.H.V.); 2 Department of Biology, School of Sciences, University of Minho, Campus de Gualtar, Braga 4710-057, Portugal; 3 Mountain Research Center (CIMO), ESA, Polytechnic Institute of Bragança, Apartado 1172, Bragança 5301-855, Portugal; 4 Department of Plant Physiology, Institute for Biological Research “Siniša Stanković”, University of Belgrade, 11000 Belgrade, Serbia; E-Mail: mris@ibiss.bg.ac.rs; 5 Plant Research International, Wageningen University and Research Centre, 6700AA Wageningen, The Netherlands; E-Mail: leo.vangriensven@wur.nl; 6 i3S-Instituto de Investigação e Inovação em Saúde, Universidade do Porto, Porto, Portugal; 7 Cancer Drug Resistance Group, Institute of Molecular Pathology and Immunology of the University of Porto, IPATIMUP, Rua Júlio Amaral de Carvalho, 45, Porto 4200-135, Portugal; 8 Department of Pathology and Oncology, Faculty of Medicine, the University of Porto, Alameda Prof. Hernâni Monteiro, Porto 4200-319, Portugal

**Keywords:** *Cordyceps militaris* methanolic extract, p53, p21, cell cycle arrest, DNA damage

## Abstract

*Cordyceps militaris* (L.) Link, an edible entomopathogenic fungus widely used in traditional Chinese medicine, has numerous potential medicinal properties including antitumor activity. The methanolic extract of *C. militaris* fruiting body was recently shown to have tumor cell growth inhibitory activity in several human tumor cell lines. Nonetheless, the mechanism of action involved is still not known. This work aimed at further studying the effect of the methanolic extract of *C. militaris* regarding its antitumor mechanism of action, using the non-small cell lung cancer cell line (NCI-H460) as a model. Results showed that treatment with the extract decreased cellular proliferation, induced cell cycle arrest at G0/G1 and increased apoptosis. In addition, the extract increased the levels of p53 and p21. Moreover, an increase in p-H2A.X and 53BP1 levels, together with an increase in the number of 53BP1 foci/cell (all indicative of DNA damage), were also observed after treatment with the extract. This work suggests that this extract affected NCI-H460 cellular viability through a mechanism involving DNA damage and p53 activation. This further supports the potential of this extract as a source of bioactive compounds, which may be used in anticancer strategies.

## 1. Introduction

*Cordyceps militaris* (L.) Link, an edible Ascomycete, is an entomopathogenic fungus widely used in traditional Chinese medicine [1]. Indeed, it has been described as having numerous potent medicinal properties such as antitumor, anti-oxidant and anti-inflammatory [1,2].

Several studies, both *in vitro* as well as in human tumor xenografts in mice, refer to the antitumor activity of *C. militaris* [3,4]. The majority of these studies have been mainly carried out with water extracts [5,6,7,8,9] or with isolated compounds (namely cordycepin) [10,11].

On the other hand, information on the antitumor potential of methanolic extracts of *C. militaris* is still scarce, even though such activity has previously been described for the methanolic extracts of *C. sinensis* (a mushroom from the *Cordyceps* genus that is relatively similar to *C. militaris*, both at the biological as well at the chemical levels), in different tumor cell lines [1,12,13,14]. A previous study from some of us has shown that a methanolic extract of *C. militaris* fruiting body presented activity as inhibitor of cell growth towards a panel of human tumor cell lines (while not affecting the proliferation of non-tumor porcine liver primary cells). This effect was further confirmed in other human tumor cell lines, in a recently published work in which the composition and antitumor activity of methanolic extracts from the mycelia and fruiting body of *C. militaris* were compared [15]. However, to date, there is no information regarding the mechanism of action of this extract.

Therefore, the present study aimed at understanding the mechanism of action of a methanolic extract of *C. militaris*, particularly its effect in cellular proliferation, cell cycle, apoptosis and DNA damage, using the NCI-H460 non-small cell lung cancer cell line as a model.

## 2. Results and Discussion

### 2.1. Effect of C. militaris Methanolic Extract in NCI-H460 Cell Viability

We have previously shown that a methanolic extract from *C. militaris* fruiting body could inhibit cell growth in human tumor cell lines and was not toxic to non-tumor liver porcine primary cells (PLP2) [16]. Decreased cell growth following treatment with this extract was recently further observed in various other human tumor cell lines [15]. Nevertheless, the mechanism of action of this extract has never been investigated. The present study aimed at gaining insight into the mechanism of action of the extract in a non-small cell lung cancer cell line (NCI-H460). The reason for selecting this cell line is that non-small cell lung cancer (NSCLC) is still a cause for many of the deaths related to cancer and, in addition, this cell line has wild-type p53 which would allow detecting p53 dependent mechanisms of action.

**Figure 1:**
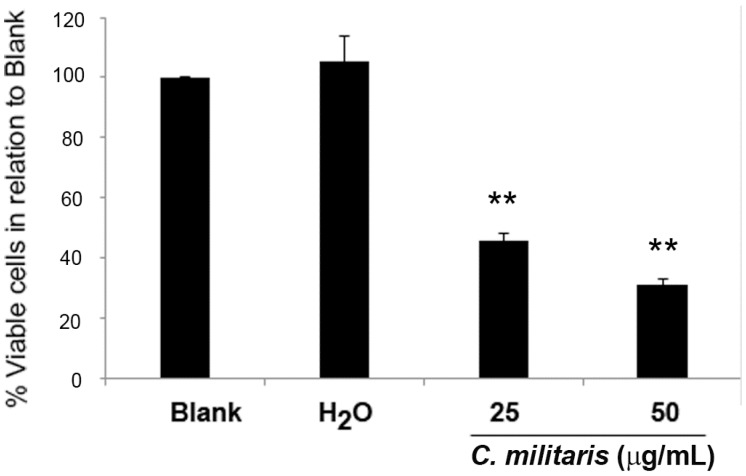
Effect of *C. militaris* methanolic fruiting body extract on NCI-H460 cell viability. Viable cell number was analyzed 48 h after incubation with complete medium (Blank), 25 μg/mL or 50 μg/mL extract or with the highest vehicle concentration (H_2_O). Results are presented as a percentage of viable cells in relation to blank cells and are the mean ± SEM of six independent experiments. ** *p* ≤ 0.001 blank *vs.* treatment.

The GI_50_ concentration of the methanolic extract of *C. militaris* had been previously found to be 47.8 μg/mL for the NCI-H460 cells, using the sulforhodamine B (SRB) assay [16]. To further confirm this, the effect of the extract in NCI-H460 cells was analyzed in terms of cell viability, using the trypan blue exclusion assay. To observe a possible dose/response effect, two concentrations were selected, 50 μg/mL (corresponding to the GI_50_ concentration) and 25 μg/mL (corresponding to half the GI_50_ concentration). Results showed that the extract significantly decreased the NCI-H460 cell viability in a dose-dependent manner. Indeed, treatment with 25 μg/mL of extract reduced cell viability to 42.3% ± 1.2% (in relation to blank cells) and treatment with 50 μg/mL further decreased cell viability to 28.0% ± 3.1% (Figure 1).

As expected, the vehicle (H_2_O) presented no effect on cell viability. From these results, we concluded that treatment with 50 μg/mL (GI_50_) caused a greater reduction of cell viability than expected (since the GI_50_ concentration reduces cell growth by 50%). The observed discrepancy between the GI_50_ concentration determined with the SRB assay and the determination of viable cell number with that same concentration is not abnormal in these types of studies and may be justified by the differences in the methodologies used in both studies. Indeed, the SRB assay (which was carried out in the study by Reis *et al.* to determine the GI_50_ concentration [16]) was carried out in 5% FBS culture medium, whereas all the assays carried out in the present study were performed in 10% FBS supplemented medium.

To assess the mechanisms involved in the decrease in cell viability induced by treatment with the extract, cell cycle profile and apoptosis were then analyzed.

### 2.2. Effect of the Extract on NCI-H460 Cellular Proliferation and Cycle Profile

**Figure 2:**
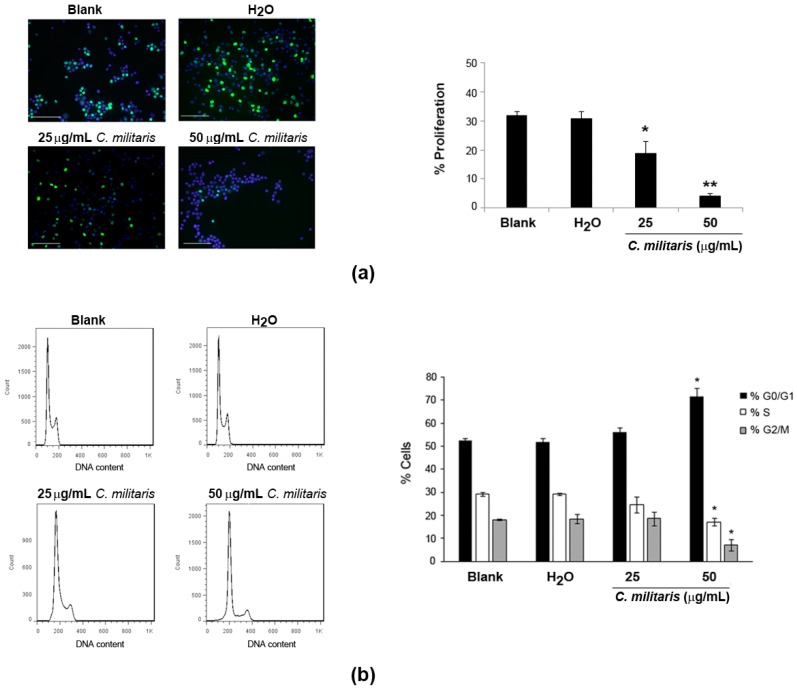
Effect of *C. militaris* methanolic fruiting body extract on NCI-H460 cellular proliferation (**a**); and cell cycle distribution (**b**). Cells were treated for 48 h with complete medium (Blank), 25 μg/mL or 50 μg/mL extract or with the highest vehicle (H_2_O) concentration. (**a**) Left panel: Representative fluorescence microscopy images of BrdU incorporation (**green**); and DAPI stained nuclei (**blue**). Bar corresponds to 20 μm. Right panel: % of BrdU-incorporating cells; (**b**) Left panel: Representative cell cycle histograms. Right panel: Distribution of cells in cell cycle phase. Results are the mean ± SEM of, at least, three independent experiments. * *p* ≤ 0.05 and ** *p* ≤ 0.001 blank *vs.* treatment.

To understand if the extract was affecting proliferation of NCI-H460 cells, BrdU incorporation was analyzed after 48 h treatment of cells with the extract. Results revealed a significant and dose-dependent decrease in the levels of proliferation following treatment with the extract (Figure 2a). This was observed by a reduction in the percentage of BrdU-incorporating cells from 32.0% (Blank) to 18.7% following treatment with 25 μg/mL, which was further reduced to 4.1% following treatment with the 50 μg/mL concentration.

These results indicated that the extract might be affecting the normal cell cycle progression. Therefore, the cell cycle profile of NCI-H460 cells treated with the extract was analyzed by flow cytometry. This allows to easily estimate the percentage of cells in the different phases of the cell cycle [17]. Results showed clear alterations in the cell cycle profile of NCI-H460 cells following treatment with the extract, which were however only considered to be statistically significant with the highest concentration (50 μg/mL; Figure 2b). Indeed, treatment with this concentration of extract (50 μg/mL) caused an increase in the percentage of cells at the G0/G1 phase from 52.6% (Blank) to 71.7%. This was accompanied by a decrease in percentage of cells in both the S and the G2/M phases. Indeed, a decrease in the S phase from 29.1% (Blank) to 17.1% (following treatment) was observed, as well as a decrease in the G2/M phase from 18.1% (Blank) to 7.0% (following treatment).

Interestingly, a previous study using *C. militaris* and its mycelial fermentation has shown a similar effect in human glioblastoma cells, with both fractions arresting glioblastoma GBM8401 cells in the G0/G1 phase [18]. Nevertheless, in that same study, an arrest at the G2/M phase was also observed with the same extracts but in a different cell line (U-87MG cells). This suggests that the effect of the extract might depend on the genetic background of the tumor cells being treated [18]. Of interest in this context is another study in which cordycepin (an isolated compound from *Cordyceps* species, which seems to be present in the methanolic extract of *C. militaris* [15]) inhibited cell cycle progression of colorectal cancer cells at the G0/G1 phase [19]. We do not know which compounds are responsible for the cell cycle arrest in G0/G1 verified in the present study, but it is possible that cordycepin might be involved in such activity. Further studies aiming at confirming the presence of cordycepin in the studied extract and evaluating the effect of cordycepin in NCI-H460 cells, would allow further exploration of this possibility.

### 2.3. Effect of the Extract on NCI-H460 Cellular Apoptosis

The decrease in cellular viability that had been observed following treatment of NCI-H460 cells with the extract could also be due to increased cell death. Thus, the effect of the methanolic extract (25 and 50 µg/mL) on apoptosis was also investigated. Preliminary results showed a decrease in total PARP levels analyzed by Western blot (data not shown), which might indicate that PARP was being cleaved (considered a useful hallmark of apoptosis). To confirm the involvement of apoptosis in the mechanism of action of the extract, a specific apoptosis assay was used consisting in flow cytometric analysis of cells after labeling them with Annexin V-FITC/PI. Results showed that the extract induced apoptosis in cells treated with either of the concentrations being tested (Table 1). This was observed by a statistically significant increase in the levels of early apoptosis from 5.9% (in Blank cells) to 13.7% and 21.4% (in NCI-H460 cells treated with 25 µg/mL or 50 µg/mL of the extract, respectively).

**Table 1:** Apoptosis levels in NCI-H460 cells treated with *C. militaris* fruiting body methanolic extract.

		% Apoptosis	
		Early	Late
**Blank**		5.9% ± 1.2%	1.5% ± 0.8%
**H_2_O**		6.3% ± 2.0%	2.2% ± 0.5%
***C. militaris***	25 µg/mL	13.7% ± 1.4% *	3.4% ± 0.3%
	50 µg/mL	21.4% ± 4.2% *	5.0% ± 5.3%

Results are the mean ± SEM of three independent experiments. * *p* ≤ 0.05 blank *vs.* treatment.

Several studies have previously referred the apoptotic activity of other types of *C. militaris* extracts (namely aqueous extracts) in human tumor cell lines, including human leukemia cells [6,9], human breast cancer cells [8] and human lung carcinoma cells [20]. Interestingly, some of the *C. militaris*’isolated compounds, namely cordycepin (described as being present in another methanolic extract of *C. militaris* fruiting body [15]), have shown activity as apoptosis inducers in several human tumor cells [10,11,21].

### 2.4. Effect of the Extract in p53 and p21 Protein Levels in NCI-H460 Cells

**Figure 3:**
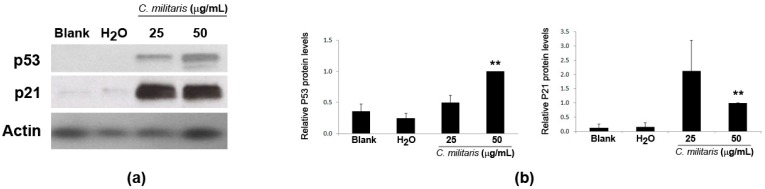
Expression of p53 and p21 in NCI-H460 cells following treatment with *C. militaris* methanolic fruiting body extract. Cells were treated for 48 h with complete medium (Blank), 25 μg/mL or 50 μg/mL of the extract or with the highest vehicle (H_2_O) concentration. Actin was used as a loading control. (**a**) Representative Western blot images of, at least, three independent experiments; (**b**) Densitometry analysis of the Western blots. Results are the mean ± SEM and are expressed after normalization of the values obtained for each protein with the values obtained for actin and further expressed in relation to treatment with 50 μg/mL extract. ** *p* < 0.01 blank *vs.* treatment.

Given that the previous results showed that the methanolic extract caused cell cycle arrest (at G0/G1 phase) and induced apoptosis in NCI-H460 cells, it was then decided to analyze the expression levels of p53 and p21 proteins in NCI-H460 cells following treatment with the extract. Results from the Western blot analysis clearly showed an increase in the expression of p53 protein following treatment with the extract. In addition, the levels of p21 (Figure 3) (a p53 transcriptional target [22] described as being a G1-checkpoint cyclin-dependent kinase inhibitor [23]), were also found to increase, supporting the idea that this extract exerts its function via p53.

There has been increasing interest in finding strategies to increase p53’s activity in cancer therapy; p53 is considered the guardian of the genome and its activation is known to result in cell cycle delay (to allow DNA repair) and also to trigger cell death by apoptosis [24]. About 50% of all human cancers harbor mutations or loss of p53 expression [25,26], and even wild-type (wt) p53 cancers may also have downstream members of p53 regulatory signaling affected, which may lead to disruption in p53 functions [27]. Therefore, strategies which result in p53 activation and of its targets could result in p53-dependent increase in cell death and cell cycle arrest, ultimately affecting tumor development in wt p53 tumors.

### 2.5. Effect of the Extract in DNA Damage of NCI-H460 Cells

**Figure 4:**
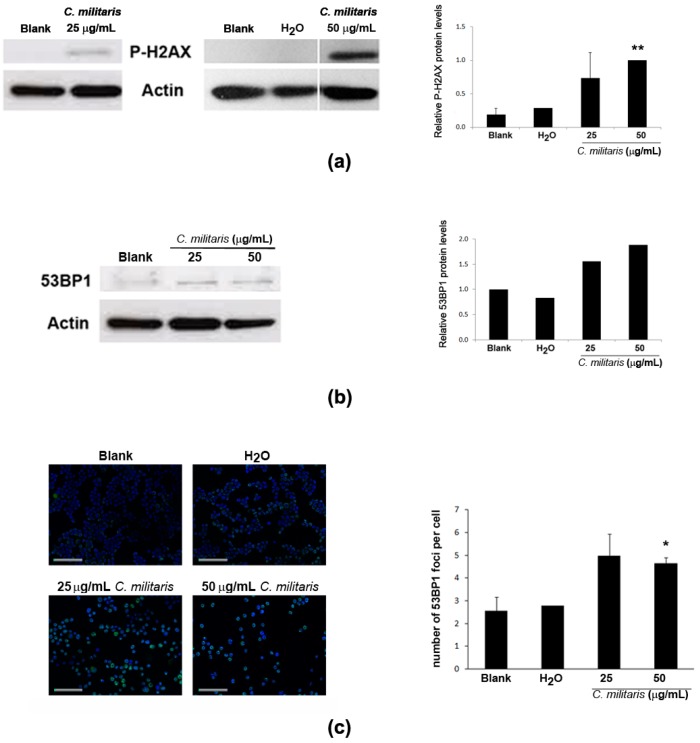
Effect of treatment with *C. militaris* methanolic fruiting body extract in NCI-H460 cellular DNA damage. Cells were treated for 48 h with complete medium (Blank), 25 μg/mL or 50 μg/mL extract or with the highest vehicle (H_2_O) concentration. (**a**) Analysis of the expression of P-H2AX by Western blot. Actin was used as loading control. Left Panel: Representative images. Right Panel: Densitometry analysis of the Western blots. Results are expressed after normalization of the P-H2AX values with the values for actin and further expressed in relation to treatment with 50 μg/mL extract. Results are the mean ± SEM of, at least, three independent experiments (except for solvent control (H_2_O) which results from two experiments only); (**b**) Analysis of the expression of 53BP1 by Western blot. Actin was used as loading control. Left Panel: Representative images. Right Panel: Densitometry analysis of the Western blots. Results are expressed after normalization of the 53BP1values with the values for actin and further expressed in relation to blank. Results are the mean of two experiments only; (**c**) Analysis of the number of 53BP1 foci per cell. Left Panel: Representative fluorescence microscopy images of 53BP1 foci (**green**) and DAPI stained nuclei (**blue**). Bar corresponds to 20 μm. Right Panel: Quantification of 53BP1 foci. Results are the mean ± SEM of three independent experiments (except for the control (H_2_O), which results from two experiments only). * *p* ≤ 0.05 and ** *p* ≤ 0.01 blank *vs.* treatment.

Activation of p53 may result from DNA damage [22,28]. Therefore, to assess if the extract was causing DNA damage in NCI-H460 cells, the phosphorylation of H2A.X (a highly specific and sensitive molecular marker for DNA damage [29]) was analyzed in this study. Results showed an increase in the levels of p-H2A.X following treatment with the extract (Figure 4a). Moreover, the levels of 53BP1 protein, an important mediator of the DNA damage response [30,31], were also found to increase after treatment with the extract (Figure 4b), together with an increase in its distribution at nuclear foci (Figure 4c), typical of cellular response to DNA damage [32]. Overall, these results suggest that the methanolic extract of *C. militaris* induces DNA damage.

In conclusion, our results showed that the methanolic extract of *C. militaris* at the 25 μg/mL concentration significantly inhibited cell proliferation and induced apoptosis of NCI-H460 cells. However, this concentration did not significantly inhibited p53 and p21 expression and DNA damage (even though statistically significant increases in the concentrations of these proteins were verified with 50 μg/mL). These results suggest that *C. militaris* may have other intracellular mechanisms leading to apoptosis of NCI-H460 cells. This will be investigated in future work.

## 3. Experimental Section

### 3.1. Extract

The methanolic extract used in this study was obtained from cultivated fruiting bodies of *Cordyceps militaris* (L.) Link (strain: MCI 10304, Meshtech Cordyceps Institute, Bangkok, Thailand), as published in [16]. The chemical characterization of the studied extract is provided in the same publication. Briefly, lyophilized sample was extracted by stirring with methanol for 1 h and filtered through Whatman No. 4 paper. The residue was then extracted with methanol for 1 h. The combined methanolic extracts were evaporated at 40 °C to dryness. A stock solution at 8 mg/mL in H_2_O was kept at −20 °C.

### 3.2. Cell Culture

The non-small cell lung cancer cell line NCI-H460 (a kind gift from NCI, Bethesda, MD, USA) was maintained in RPMI-1640 medium with UltraGlutamine I and 25 mM Hepes (Lonza, Basel, Switzerland) supplemented with 10% FBS (Biowest, Nuaillé, France) at 37 °C in a humidified incubator with 5% CO_2_ in air. Cell number and viability were assessed with Trypan blue exclusion assay. All experiments were carried out with exponentially growing cells (presenting more than 90% cell viability).

### 3.3. Cell Treatments with C. militaris Extract

Cells (1 × 10^5^ cells/well) were plated in 6-well plates and incubated for 24 h at 37 °C in 5% CO_2_ to allow cell adhesion. Cells were then treated for 48 h with 25 μg/mL or 50 μg/mL of the extract for 48 h. Cells were also treated with complete medium (Blank) or with the corresponding concentrations of the extract solvent (H_2_O, Control). Following treatment, cells were processed according to different protocols, as follows.

#### 3.3.1. Analysis of Cell Cycle Profile by Flow Cytometry

Following 48 h treatment, cell pellets were fixed in ice-cold 70% ethanol for at least 12 h. Cells were then resuspended in 5 μg/mL propidium iodide and 0.1 mg/mL RNase A in PBS. Cellular DNA content was analyzed using a FACSCalibur flow cytometer (BD Biosciences). The percentage of cells in the different phases of cell cycle (G0/G1, S and G2/M) was determined by plotting at least 20,000 events per sample, after excluding cell debris and aggregates [33], using the FlowJo 7.6.5 software (Tree Star, Inc., Ashland, OR, USA).

#### 3.3.2. Analysis of Cellular Proliferation with the BrdU Incorporation Assay

Following 47 h treatment, cells were incubated for 1 h with 10 μM 5-bromo-2′-deoxyuridine (BrdU, Sigma-Aldrich) at 37 °C and 5% CO_2_. Cells were then washed, fixed in 4% paraformaldehyde and cytospins prepared. Following a DNA denaturation step consisting of 20 min incubation in 2 M HCl at room temperature, cells were incubated with mouse anti-BrdU antibody (1:10; Dako) for 1 h at room temperature and further incubated in the dark with anti-mouse-Ig-FITC (1:100; Dako, Glostrup, Denmark) for 30 min. Cells were then washed again twice with PBS-T-B and slides were prepared with Vectashield mounting medium with DAPI (Vector Laboratories Inc, Burlingame, CA, USA). BrdU incorporation was analyzed using a fluorescence microscope (Leica DM2000) and the percentage of BrdU incorporating cells determined by a counting at least 500 cells for each condition [34].

#### 3.3.3. Analysis of Apoptosis by Flow Cytometry

Following 48 h treatment, analysis of apoptotic cell death was carried out using the “Annexin V-FITC Apoptosis Detection kit” (Bender MedSystems, Vienna, Austria) as previously described [35]. Cells were analyzed by flow cytometry in a FACSCalibur flow cytometer (BD Biosciences, San Jose, CA, USA). All data was analyzed using the FlowJo 7.6.5 software (Tree Star, Inc., Ashland, OR, USA), plotting at least 20,000 events per sample.

#### 3.3.4. Analysis of Protein Expression by Western Blot

Following 48 h treatment with the extract, total protein lysates were prepared by lysing cell pellets in Winman’s Buffer (1% NP-40, 0.1 M Tris-HCl pH 8.0, 0.15 M NaCl and 5 mM EDTA). Total protein content was quantified using the “DC Protein assay kit” (Bio-Rad) and protein (30 µg) was subjected to SDS-PAGE (12% Bis-Tris gel). Following electrophoretic transfer onto nitrocellulose membranes (GE Healthcare), membranes were incubated with the following primary antibodies: Mouse anti-p53 (1:200; Thermo-Scientific, Waltham, MA, USA), mouse anti-p21 (1:250; Calbiochem, San Diego, CA, USA), mouse anti-53BP1 (1:400; Santa Cruz Biotechnology), *p*-Histone H2A.X (Ser 139) (1:200; Santa Cruz Biotechnology Inc, Heidelberg, Germany) and goat anti-Actin (1:2000; Santa Cruz Biotechnology). The following secondary antibodies were then used: Anti-mouse IgG-HRP; anti-rabbit IgG-HRP or anti-goat IgG-HRP (all diluted 1: 2000, Santa Cruz Biotechnology). Signal was detected with the ECL Western blot Detection kit (GE Healthcare, Buckinghamshire, UK), Hyperfilm ECL (GE Healthcare) and the Kodak GBX developer and fixer (Sigma) [36,37]. The intensity of the bands obtained in each film was quantified using the Quantity One software (BioRad, Hercules, CA, USA).

#### 3.3.5. Analysis of 53BP1 Expression by Immunofluorescence

Following 48 h treatment with the extract, cells were fixed in 4% paraformaldehyde and cytospins were prepared. Cells were incubated for 10 min in 50 mM NH_4_Cl in PBS and permeabilized in ice-cold 0.2% Triton X-100 in PBS for 10 min. After a blocking step with 2% BSA in PBS for 20 min, cells were incubated with rabbit 53BP1 antibody (Santa Cruz Biotechnology) diluted 1:200 in 2% BSA in PBS, overnight at 4 °C. Cells were then washed with 2% BSA in PBS and further incubated with rabbit-IgG-FITC antibody (1:100; Dako) for 1 h, at room temperature. Slides were mounted in Vectashield Mounting Media with DAPI (Vector Laboratories Inc, Burlingame, CA, USA) and cell images were taken using a fluorescence microscope ZEISS Axio Imager.Z1 coupled with ApoTome Imaging System microscope. Exported images in TIFF format were decompressed with Irfanview (ver. 4.35, Irfan Skiljan, Vienna, Austria) and analyzed with ImageJ software (version 1.46r). This was carried out using a program written by Dr. Niklas Schultz (GMT Department, Stockholm University, Sweden [38]) which analyses DAPI and FITC channels independently, detects cell nuclei and registers foci parameters within the nuclear area. Different parameters such as foci area, intensity and the number of foci per nucleus were given in the output file [39].

### 3.4. Statistical Analysis

All presented data results were obtained from at least three independent experiments (unless otherwise stated in the results section). All data was statistically analyzed with the two-tailed paired Student’s *t*-test (except for the data presented in Figure 2b and in Table 1, which were analyzed with the unpaired Student’s *t*-test). Results were considered statistically significant when *p* ≤ 0.05.

## 4. Conclusions

This study showed that a methanolic extract of *Cordyceps militaris* fruiting body reduced the number of viable NCI-H460 human tumor cells by decreasing cellular proliferation, increasing the number of cells in the G0/G1 phase of the cell cycle and inducing cell death by apoptosis. Moreover, this extract was shown to probably induce DNA damage and to increase the cellular levels of p53 and p21. This is particularly interesting since p53 is an attractive therapeutic target and molecules causing an increase in p53 wt levels may contribute to the development of anticancer therapies. However, in order to further confirm the mechanism of action of this extract further studies would be required, namely studies on the effect of this extract on NCI-H460 cells following p53 silencing and on other tumor cell lines without p53 or with a p53 mutation. Furthermore, additional studies on the effect of compounds isolated from this extract would also be important. Indeed, the chemical characterization of this extract has already allowed the identification of *p*-hydroxybenzoic and cinnamic acids [16]. In addition, cordycepin (an isolated compound from *Cordyceps* species) seems to be present in methanolic extract of *C. militaris* [15]. The effect of all these compounds has been previously studied in NCI-H460 cells, with both cordycepin and cinnamic acid showing *in vitro* cell growth inhibitory activity [40,41]. Nevertheless, when addressing the possibility of using extracts/isolated compounds *in vivo*, extrapolation of the results obtained using cell line models must be carefully analyzed. Pharmacokinetic studies, as well as the optimization of treatment conditions, are needed in order to be successful. Overall, this work further supports the potential of *C. militaris* extracts in the search for bioactive compounds, which may be of use in anticancer strategies.

## References

[1] Das S.K., Masuda M., Sakurai A., Sakakibara M. (2010). Medicinal uses of the mushroom *Cordyceps militaris*: Current state and prospects. Fitoterapia.

[2] Park N.S., Lee K.S., Sohn H.D., Kim D.H., Lee S.M., Park E., Kim I., Je Y.H., Jin B.R. (2005). Molecular cloning, expression, and characterization of the Cu, Zn superoxide dismutase (SOD1) gene from the entomopathogenic fungus *Cordyceps militaris*. Mycologia.

[3] Park S.E., Kim J., Lee Y.W., Yoo H.S., Cho C.K. (2009). Antitumor activity of water extracts from *Cordyceps militaris* in NCI-H460 cell xenografted nude mice. J. Acupunct. Meridian Stud..

[4] Yoo H.S., Shin J.W., Cho J.H., Son C.G., Lee Y.W., Park S.Y., Cho C.K. (2004). Effects of *Cordyceps militaris* extract on angiogenesis and tumor growth. Acta Pharmacol. Sin..

[5] Yue K., Ye M., Zhou Z., Sun W., Lin X. (2013). The genus Cordyceps: A chemical and pharmacological review. J. Pharm. Pharmacol..

[6] Park C., Hong S.H., Lee J.Y., Kim G.Y., Choi B.T., Lee Y.T., Park D.I., Park Y.M., Jeong Y.K., Choi Y.H. (2005). Growth inhibition of U937 leukemia cells by aqueous extract of *Cordyceps militaris* through induction of apoptosis. Oncol. Rep..

[7] Lim H., Kwon Y., Cho S., Kim J., Yoon G., Lee S., Kim H. (2004). Antitumor activity of *Cordyceps militaris* on human cancer cell line. Korean J. Pharmacogn..

[8] Jin C.Y., Kim G.Y., Choi Y.H. (2008). Induction of apoptosis by aqueous extract of *Cordyceps militaris* through activation of caspases and inactivation of Akt in human breast cancer MDA-MB-231 Cells. J. Microbiol. Biotechnol..

[9] Lee H., Kim Y.J., Kim H.W., Lee D.H., Sung M.K., Park T. (2006). Induction of apoptosis by *Cordyceps militaris* through activation of caspase-3 in leukemia HL-60 cells. Biol. Pharm. Bull..

[10] Baik J.S., Kwon H.Y., Kim K.S., Jeong Y.K., Cho Y.S., Lee Y.C. (2012). Cordycepin induces apoptosis in human neuroblastoma SK-N-BE(2)-C and melanoma SK-MEL-2 cells. Indian J. Biochem. Biophys..

[11] Tuli H.S., Sharma A.K., Sandhu S.S., Kashyap D. (2013). Cordycepin: A bioactive metabolite with therapeutic potential. Life Sci..

[12] Shrestha B., Zhang W., Zhang Y., Liu X. (2012). The medicinal fungus *Cordyceps militaris*: Research and development. Mycol. Prog..

[13] Rao Y.K., Fang S.H., Tzeng Y.M. (2007). Evaluation of the anti-inflammatory and anti-proliferation tumoral cells activities of *Antrodia camphorata*, *Cordyceps sinensis*, and *Cinnamomum osmophloeum* bark extracts. J. Ethnopharmacol..

[14] Huang H., Wang H., Luo R.C. (2007). Inhibitory effects of cordyceps extract on growth of colon cancer cells. Zhong Yao Cai J. Chin. Med. Mater..

[15] Liu X., Huang K., Zhou J. (2014). Composition and Antitumor Activity of the Mycelia and Fruiting Bodies of *Cordyceps militaris*. J. Food Nutr. Res..

[16] Reis F.S., Barros L., Calhelha R.C., Ciric A., van Griensven L.J., Sokovic M., Ferreira I.C. (2013). The methanolic extract of *Cordyceps militaris* (L.) Link fruiting body shows antioxidant, antibacterial, antifungal and antihuman tumor cell lines properties. Food Chem.Toxicol. Int. J. Publ. Br. Ind. Biol. Res. Assoc..

[17] Jayat C., Ratinaud M.H. (1993). Cell cycle analysis by flow cytometry: Principles and applications. Biol. Cell Auspices Eur. Cell Biol. Organ..

[18] Yang C.H., Kao Y.H., Huang K.S., Wang C.Y., Lin L.W. (2012). *Cordyceps militaris* and mycelial fermentation induced apoptosis and autophagy of human glioblastoma cells. Cell Death Dis..

[19] He W., Zhang M.F., Ye J., Jiang T.T., Fang X., Song Y. (2010). Cordycepin induces apoptosis by enhancing JNK and p38 kinase activity and increasing the protein expression of Bcl-2 pro-apoptotic molecules. J. Zhejiang Univ. Sci. B.

[20] Park S.E., Yoo H.S., Jin C.Y., Hong S.H., Lee Y.W., Kim B.W., Lee S.H., Kim W.J., Cho C.K., Choi Y.H. (2009). Induction of apoptosis and inhibition of telomerase activity in human lung carcinoma cells by the water extract of *Cordyceps militaris*. Food Chem. Toxicol. Int. J. Publ. Br. Ind. Biol. Res. Assoc..

[21] Jeong J.W., Jin C.Y., Park C., Hong S.H., Kim G.Y., Jeong Y.K., Lee J.D., Yoo Y.H., Choi Y.H. (2011). Induction of apoptosis by cordycepin via reactive oxygen species generation in human leukemia cells. Toxicol. Int. J. Publ. Assoc. BIBRA.

[22] Lakin N.D., Jackson S.P. (1999). Regulation of p53 in response to DNA damage. Oncogene.

[23] Abukhdeir A.M., Park B.H. (2008). p21 and p27: Roles in carcinogenesis and drug resistance. Expert Rev. Mol. Med..

[24] Amundson S.A., Myers T.G., Fornace A.J. (1998). Roles for p53 in growth arrest and apoptosis: Putting on the brakes after genotoxic stress. Oncogene.

[25] Soussi T., Dehouche K., Beroud C. (2000). p53 website and analysis of p53 gene mutations in human cancer: Forging a link between epidemiology and carcinogenesis. Hum. Mutat..

[26] Vassilev L.T. (2005). p53 Activation by small molecules: Application in oncology. J. Med. Chem..

[27] Athar M., Elmets C.A., Kopelovich L. (2011). Pharmacological activation of p53 in cancer cells. Curr. Pharm. Des..

[28] Kuribayashi K., El-Deiry W.S. (2008). Regulation of programmed cell death by the p53 pathway. Adv. Exp. Med. Biol..

[29] Mah L.J., El-Osta A., Karagiannis T.C. (2010). gammaH2AX: A sensitive molecular marker of DNA damage and repair. Leukemia.

[30] Wang B., Matsuoka S., Carpenter P.B., Elledge S.J. (2002). 53BP1, a mediator of the DNA damage checkpoint. Science.

[31] Mallette F.A., Richard S. (2012). K48-linked ubiquitination and protein degradation regulate 53BP1 recruitment at DNA damage sites. Cell Res..

[32] Fadlalla K., Watson A., Yehualaeshet T., Turner T., Samuel T. (2011). Ruta graveolens extract induces DNA damage pathways and blocks Akt activation to inhibit cancer cell proliferation and survival. Anticancer Res..

[33] Abreu R.M., Ferreira I.C., Calhelha R.C., Lima R.T., Vasconcelos M.H., Adega F., Chaves R., Queiroz M.J. (2011). Anti-hepatocellular carcinoma activity using human HepG2 cells and hepatotoxicity of 6-substituted methyl 3-aminothieno[3,2-*b*]pyridine-2-carboxylate derivatives: *In vitro* evaluation, cell cycle analysis and QSAR studies. Eur. J. Med. Chem..

[34] Preto A., Goncalves J., Rebocho A.P., Figueiredo J., Meireles A.M., Rocha A.S., Vasconcelos H.M., Seca H., Seruca R., Soares P. (2009). Proliferation and survival molecules implicated in the inhibition of BRAF pathway in thyroid cancer cells harbouring different genetic mutations. BMC Cancer.

[35] Queiroz M.J., Peixoto D., Calhelha R.C., Soares P., dos Santos T., Lima R.T., Campos J.F., Abreu R.M., Ferreira I.C., Vasconcelos M.H. (2013). New di(hetero)arylethers and di(hetero)arylamines in the thieno[3,2-b]pyridine series: Synthesis, growth inhibitory activity on human tumor cell lines and non-tumor cells, effects on cell cycle and on programmed cell death. Eur. J. Med. Chem..

[36] Oliveira M., Reis F.S., Sousa D., Tavares C., Lima R.T., Ferreira I.C., Santos T.D., Vasconcelos M.H. (2014). A methanolic extract of *Ganoderma lucidum* fruiting body inhibits the growth of a gastric cancer cell line and affects cellular autophagy and cell cycle. Food Funct..

[37] Lima R.T., Martins L.M., Guimaraes J.E., Sambade C., Vasconcelos M.H. (2006). Chemosensitization effects of XIAP downregulation in K562 leukemia cells. J. Chemother..

[38] Markova E., Schultz N., Belyaev I.Y. (2007). Kinetics and dose-response of residual 53BP1/gamma-H2AX foci: Co-localization, relationship with DSB repair and clonogenic survival. Int. J. Radiat. Biol..

[39] Seca H., Lima R.T., Almeida G.M., Sobrinho-Simoes M., Bergantim R., Guimaraes J.E., Vasconcelos M.H. (2014). Effect of MiR-128 in DNA Damage of HL-60 Acute Myeloid Leukemia Cells. Curr. Pharm. Biotechnol..

[40] Aramwit P., Bang N., Ratanavaraporn J., Nakpheng T., Srichana T. (2014). An Anti-Cancer Cordycepin Produced by *Cordyceps militaris* Growing on the Dead Larva of Bombyx mori Silkworm. J. Agric. Sci..

[41] Vaz J.A., Almeida G.M., Ferreira D.F., Ferreira I.C.F.R., Martins A., Vasconcelos M.H. (2012). *Clitocybe alexandri* extract induces cell cycle arrest and apoptosis in a lung cancer cell line: Identification of phenolic acids with cytotoxic potential. Food Chem..

